# Direct observation of secondary nucleation along the fibril surface of the amyloid *β* 42 peptide

**DOI:** 10.1073/pnas.2220664120

**Published:** 2023-06-12

**Authors:** Dev Thacker, Mohammad Barghouth, Mara Bless, Enming Zhang, Sara Linse

**Affiliations:** ^a^Department of Biochemistry and Structural Biology, Lund University, 22100 Lund, Sweden; ^b^Department of Clinical Sciences in Malmö, Lund University Diabetes Centre, Lund University, 22100 Lund, Sweden; ^c^Department of Chemistry and Applied Biosciences, ETH Zürich, 8093 Zürich, Switzerland; ^d^NanoLund Center for NanoScience, Lund University, 22100 Lund, Sweden

**Keywords:** amyloid aggregation, secondary nucleation, dSTORM

## Abstract

Alzheimer’s disease is a neurodegenerative condition which involves heavy neuronal cell death linked to oligomers formed during the aggregation process of the amyloid *β* peptide 42 (A*β*42). The aggregation of A*β*42 involves both primary and secondary nucleation. Secondary nucleation dominates the generation of oligomers and involves the formation of new aggregates from monomers on catalytic fibril surfaces. Understanding the molecular mechanism of secondary nucleation may be crucial in developing a targeted cure. Here, the self-seeded aggregation of WT A*β*42 is studied using direct stochastic optical reconstruction microscopy (dSTORM) with separate fluorophores in seed fibrils and monomers. Seeded aggregation proceeds faster than nonseeded reactions because the fibrils act as catalysts. The dSTORM experiments show that monomers grow into relatively large aggregates on fibril surfaces along the length of fibrils before detaching, thus providing a direct observation of secondary nucleation and growth along the sides of fibrils. The experiments were repeated for cross-seeded reactions of the WT A*β*42 monomer with mutant A*β*42 fibrils that do not catalyze the nucleation of WT monomers. While the monomers are observed by dSTORM to interact with noncognate fibril surfaces, we fail to notice any growth along such fibril surfaces. This implies that the failure to nucleate on the cognate seeds is not a lack of monomer association but more likely a lack of structural conversion. Our findings support a templating role for secondary nucleation, which can only take place if the monomers can copy the underlying parent structure without steric clashes or other repulsive interactions between nucleating monomers.

## Significance

Alzheimer’s disease is a devastating neurodegenerative disease and the most common cause of dementia. It involves the self-assembly of the amyloid *β* peptide into fibrillar aggregates through an autocatalytic amplification mechanism—secondary nucleation. This study investigates the mechanism of surface-catalyzed secondary nucleation and asks whether it has a templating role in which nucleating species take up the structure of the parent fibril. This is important in understanding the formation of oligomers, which are considered to be the neurotoxic species during the disease. The results provide a high-resolution view of secondary nucleation and show the steps of attachment, growth along the fibrils and finally detachment and appearance of new fibrils.

**Fig. 1.**
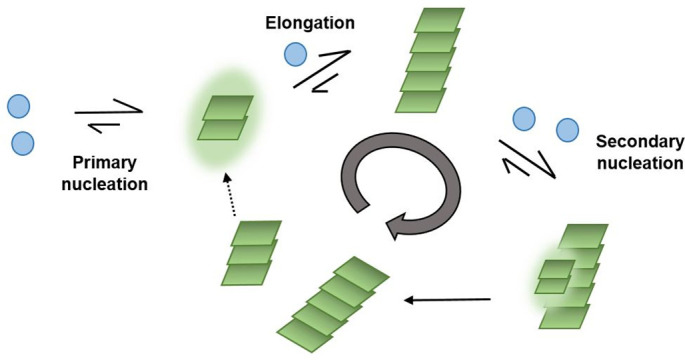
Microscopic steps involved in self-assembly of A*β*42. Primary nucleation involves the formation of nuclei (green) from monomers alone (blue). In secondary nucleation, the fibrils act as a catalytic surface for the formation of new aggregates. Elongation involves growth at fibrillar ends by monomer addition.

Aggregopathies like Alzheimer’s disease, Parkinson’s disease, and Huntington’s disease involve a series of complex misfolding steps of amyloid proteins. This makes it difficult to target the proteins involved and requires detailed knowledge of the underlying molecular events (1–3). It has been established that the plaques formed by fibrillar aggregates, which are a hallmark of these diseases, are themselves inert, but the intermediate oligomers are the neurotoxic species (4–6). The amyloid proteins involved, which play a role in Alzheimer’s disease, are the amyloid *β* peptide (A*β*42) (1, 7, 8) and the protein tau. These proteins form fibrillar aggregates, neurofibrillary tangles, and senile plaques in the brains of Alzheimer’s patients. While structures of end-stage fibrils are available based on different structure determination techniques (9–13), it is difficult to solve the structure for transient oligomeric species, making it crucial to understand the precise mechanism of their formation. Aggregation kinetic assays have provided information on the microscopic steps involved in the self-assembly of monomers into highly ordered fibrils, but there is still a void of information in understanding the precise mechanism by which nucleation from monomers is catalyzed on fibril surfaces. Specifically, the microscopic steps involved in the self-assembly of A*β*42 are primary nucleation, secondary nucleation, and elongation (14). Primary nucleation involves monomers only, while secondary nucleation involves both monomers and preexisting aggregates of the same peptide (Fig. 1). Fibrils thus increase the rate of nucleation (14). This microscopic step of aggregation leads to the formation of neurotoxic oligomers.

Understanding the mechanism of secondary nucleation at a molecular level is still elusive. It has been widely accepted that fibril growth by elongation involves a templating mechanism whereby the monomers that add to the ends replicate the structure of the monomers in the fibril to which they add a next layer. Recent studies have raised the question of whether structural propagation holds true also for secondary nucleation (15). In a previous study, we showed that the ability of A*β*42 monomers to nucleate on a fibril surface is very strongly linked to the ability of the monomers to adopt the structure of the parent fibril (16). In that study, WT A*β*42 monomers failed to nucleate on the surface of a mutant version of A*β*42, called 4S. In the 4S mutant, four hydrophobic residues Val18, Ala21, Val40, and Ala42 exposed on the fibril surface of A*β*42 are replaced with hydrophilic serine, causing the fibril to form an alternative structure that fails to catalyze the nucleation of WT monomers. A*β*42 is also known to not cross-seed with A*β*40, further hinting that nucleation events can fail to occur on fibrils having a structure that the monomers cannot adopt (17). An independent study showed that monomers of A*β*40 recognize structural features of A*β*16-22 fibrils and dock onto them, which catalyzes their assembly (18). *α*-synculein, the amyloid protein involved in Parkinson’s disease, can propagate through elongation but not secondary nucleation a fibril structure that is different from the most stable one at the particular solution condition (15).

Fluorescence-based single molecule methods have been proven to be extremely useful in understanding the processes involved in amyloid fibril formation (19–21). In this study, we use direct stochastic optical reconstruction microscopy (dSTORM) to probe the surface catalysis of A*β*42 monomers on fibrils and their failure to nucleate on the surface of 4S mutant A*β*42 fibrils. We covalently label A*β*42 with Alexa fluorophores using maleimide chemistry by introducing cysteine residues at defined positions of WT A*β*42 and 4S A*β*42 and use them to study self- and cross-seeded aggregation using dSTORM. For WT A*β*42, we use S8C as described since it is shown to have no perturbation on aggregation kinetics and can aggregate independently (22). For 4S A*β*42, we choose the 40th residue to introduce cysteine, thus creating a mutant 3S-V40C with three serine mutations—V18S, A21S, and A42S—and one cysteine mutation V40C. As shown in ref. 22 V40C causes the least perturbation on fibril morphology, which is highly desirable in studies of surface-catalyzed secondary nucleation as fibril morphology is known to directly affect catalytic properties of the seeds (16).

## Results

### Expression, Purification, and Covalent Labeling of Peptides with Alexa Fluors.

Sequence purity of the starting material is crucial for reproducible aggregation kinetics of peptides and its analysis. We thus expressed recombinant A*β*(M1-42) S8C, hereby referred to as WT* (Table 1), as is, that is, without any tags except Met0, which is required to initiate translation, and purified from inclusion bodies using ion exchange and size exclusion steps, as described before (14). This mode of expression of A*β*(M1-42) peptides requires that the peptide has low enough solubility to form inclusion bodies, which avoids the degradation of small unstructured proteins in *Escherichia coli*. We also expressed 3S-V40C (V18S+A21S+V40C+A42S), hereby referred to as 4S* (Table 1), in fusion with the self-cleavable tag NPro in the form of its EDDIE mutant, which drives the expressed fusion construct to inclusion bodies and cleaves off the fused peptide upon refolding.

**Table 1.** Nomenclature for the A *β*42constructs used in this study

Construct	Sequence of peptide
A*β*42 WT	MDAEFRHDSGYEVHHQKLAFFVEDVGSNKGAIIGLMVGGVVIA
A*β*42 WT*	MDAEFRHDCGYEVHHQKLAFFVEDVGSNKGAIIGLMVGGVVIA
A*β*42 4S	DAEFRHDSGYEVHHQKLSFFSEDVGSNKGAIIGLMVGGVSIS
A*β*42 4S*	DAEFRHDSGYEVHHQKLSFFSEDVGSNKGAIIGLMVGGVCIS

**Fig. 2.**
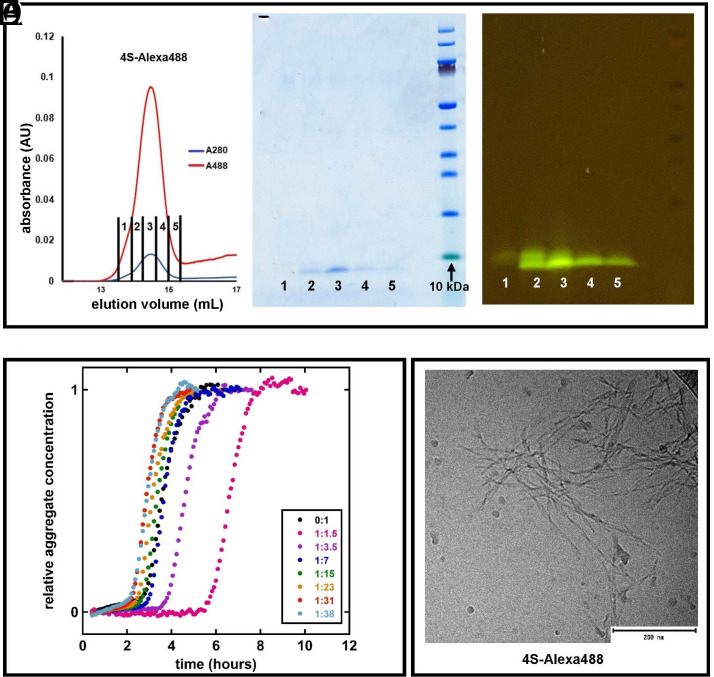
(*A*) Size exclusion chromatography of 4S* labeled with Alexa-488. The absorbance was monitored at 280 nm (blue) and 488 nm (red), which coincide, indicating efficient labeling of 4S* monomers. (*B*) SDS PAGE of the fractions collected from the SEC elution. Single bands prove that the monomers eluting are of the same molecular weight. (*C*) Fluorescence of the Alexa-488 dye on a blue-light UV table, proving that Alexa-488 has successfully bound to the 4S* monomers. In fraction 2, an extra band is seen above the normal monomer band. This might be due to heterogeneity in the peptide or the Alexa fluor. Fraction 3 was used in the subsequent experiments. (*D*) Aggregation kinetics of 4S*-Alexa488 at different ratios with unlabeled 4S followed by ThT fluorescence as a function of time in 20 mM sodium phosphate and 0.2 mM EDTA, pH 8.0, buffer. The curves shown are median values of three replicates for each ratio given as 4S*-Alexa488:4S in the inset. (*E*) cryo-TEM images of end-stage fibrils formed by 4S*-Alexa488 fibrils at a 1:1.5 molar ratio with unlabeled 4S. The scale bar represents 200 nm.

For labeling peptides with Alexa fluor maleimides, the peptides-dye mixtures were kept overnight at 4 °C and purified by size exclusion chromatography the next morning. The absorbance at 214, 280, and 488 nm was monitored. Elution peaks with absorbance at all three wavelengths were observed, indicating that all the peptide has been successfully labeled (Fig. 2*A*). The eluting peptide was collected in multiple fractions, and these were then loaded onto an SDS PAGE to confirm labeling and purity. Fig. 2*B* shows the elution fractions of Alexa-488-labeled 4S* on SDS PAGE. 4S*-Alexa488 monomers can be seen below the 10-kDa molecular weight standard in aliquots 2, 3, 4, and 5. Before staining with coomassie, the same gel was transferred to a blue-light table with an orange filter to observe fluorescence from Alexa-488 attached to the 4S monomers (Fig. 2*C*).

### Fibril Formation of 4S*-Alexa488.

In order to examine the extent of perturbation resulting from the attachment of Alexa488 to 4S* A*β*42, we studied its aggregation kinetics by ThT fluorescence and checked the morphology of fibrils using cryo-TEM.

The fibril formation of Alexa488-labeled 4S* A*β*42 was investigated under conditions at which A*β*42 is known to aggregate rapidly. Aggregation in samples with a total monomer concentration of 5 μM with different ratios of labeled peptide:unlabeled 4S was followed by monitoring ThT fluorescence as a function of time and peptide concentration at 37 °C in 20 mM sodium phosphate and 0.2 mM EDTA, pH 8.0. Under these conditions, the peptides at all ratios form ThT-positive aggregates over time. The aggregation curves have a sigmoidal-like appearance, comprising a lag phase, an exponential phase, and a final plateau, characteristic of nucleated polymerization reactions (Fig. 2*D*).

Cryo-TEM was used to study the morphology of the end-stage fibrils. In typical A*β*42 aggregates, individual filaments can be observed, and two filaments are twisted around each other along a common axis, seen as nodes that appear along the fibril at regular intervals. Fig. 2*E* shows the end-stage fibrils of the Alexa-labeled 4S* in a 1:1.5 mixture with unlabeled 4S. The same ratio of labeled:unlabeled peptide was used to make fibril seeds for the cross-seeding experiments with dSTORM.

### Self-Seeded Aggregation Kinetics Monitored by dSTORM.

To study the surface catalysis of monomers on preformed seeds (fibrils), self-seeding studies were performed between WT*-Alexa647 monomers and WT*-Alexa488 fibrils using dSTORM.

**Fig. 3.**
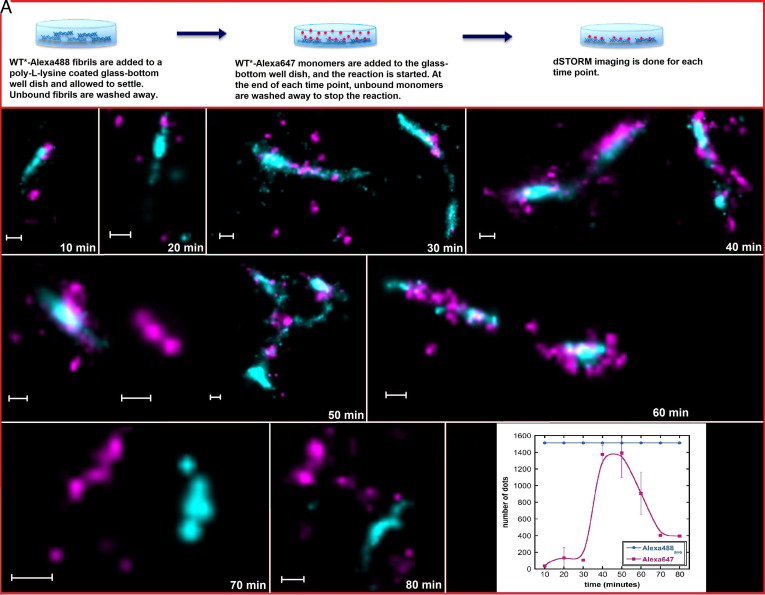
(*A*) Schematic representation of self-seeded aggregation studies to follow surface catalysis of WT* A*β*42 using dSTORM imaging. (*B*–*I*) The self-seeded aggregation reaction was followed over time, with 10-min intervals up to 80 min. WT* monomers are labeled with Alexa647 (fuchsia), and WT* fibrils are labeled with Alexa488 (cyan). The scale bars represent 200 nm. For each time point, dSTORM experiments were repeated three times. (*J*) semiquantitative analysis of Alexa647 signal derived from WT* A*β*42 monomers. Error bars represent the SD of Alexa647 signal from different image frames at each time point. Alexa488_*ave*_ represents the average number of molecules in all fibrils.

For studies of self-seeded aggregation reactions, WT*-Alexa488 fibrils (200 μL volume and 0.7 μM concentration) in 20 mM sodium phosphate and 0.2 mM EDTA, pH 8.0, buffer were deposited on a poly-lysine-coated # 1.5 (0.17 mm) glass-bottom dish. Once the fibrils were immobilized, WT*-Alexa647 monomers were added (200 μL volume and 0.7 μM concentration) to the fibrils for the reaction to begin (Fig. 3*A*). Since the reacting species here are monomers, at the end of each time point, the unbound monomers are washed away in order to stop the reaction, and the glass-bottom dish is loaded onto the dSTORM for imaging. Fig. 3 *B*–*I* shows the dSTORM images of samples taken at various time points during the self-seeding reaction of WT* A*β*42. The first time point is at 10 min, where we see WT* fibrils (cyan) and small clusters of monomers (fuchsia) on the surface of the fibrils, mainly gathered at the fibril end. At *t* = 20 min, no remarkable developments are observed, but at *t* = 30 min, significantly more clusters of monomers are seen on the surface of the fibril along its sides. These monomer clusters also appear to be larger than those found at the previous time points. At *t* = 40 min, the monomer clusters seem to have grown into larger aggregates on the fibril surface, and the fibrils are covered along their length by these aggregates. These aggregates can be seen detaching from the fibrils at *t* = 50 min, and some were also found detached as individual fibrils. In parallel, small clusters of monomers and larger aggregates can still be seen on fibril surfaces at 50 and 60 min. At the later time points, we see fibrils from both WT*-Alexa647 (fuchsia) and the original seeds of WT*-Alexa488 (cyan), indicating that the aggregation reaction has now reached the plateau and most monomers have assembled into fibrils after being catalyzed on the surface of the seeds. Notably, the original seeds now show no presence of any monomer clusters or other aggregates on their surfaces, indicating that the fibril surfaces act as catalytic surfaces for monomer assembly and proliferation, and at the end of the assembly reaction, the daughter fibrils detach from the parent seed and exist as individual fibrils.

Small dots from Alexa488 (WT* seeds) and Alexa647 (WT* monomer) signals from dSTORM images were counted using ImageJ software. Fig. 3*J* shows the average number of dots derived from fibrils in blue and from monomers near the fibril surface in pink. Each dot indicates one molecule of A*β*42. At the first three time points, the number of A*β*42 monomers at the fibril surface slowly increases. However, there is a sharp increase at the 40- and 50-min time points as these small clusters of monomers start converting into aggregates on the fibril surface, proving the catalytic nature of fibrils in proliferation of monomers. These aggregates then start detaching from the fibril surface (*t* = 60 min), and small fibrils can be observed attached to the glass-bottom dish (*t* = 70 and 80 min). This further proves that seeds act as catalysts and monomers grow along their surface before breaking away from the fibril surface, thus continuing the catalytic cycle as long as free monomers are present in solution. Note: dSTORM relies on single fluorophore count, and in a cross-*β*-sheet fibril, the fluorophores are on top of each other, resulting in superconcentration. Thus, the blinking fluorophore may overlap in position, resulting in error in the counting spots. In addition, quenching of an excited fluorophore is also a potential error. Hence, this should be considered a semiquantitative analysis.

Control studies were performed using a reverse labeling scheme, i.e., WT*-Alexa488 monomers and WT*-Alexa647 fibrils to establish that the difference in the chemical nature of the Alexa fluorophores is not an interfering factor in the interactions between the monomers and fibrils during seeded aggregation. The images from dSTORM experiments with reversed fluorophores can be seen in *SI Appendix*, Fig. S1 and show the same trend with nucleation and growth along fibrils during the process and at the end appearance of separate fibrils.

**Fig. 4.**
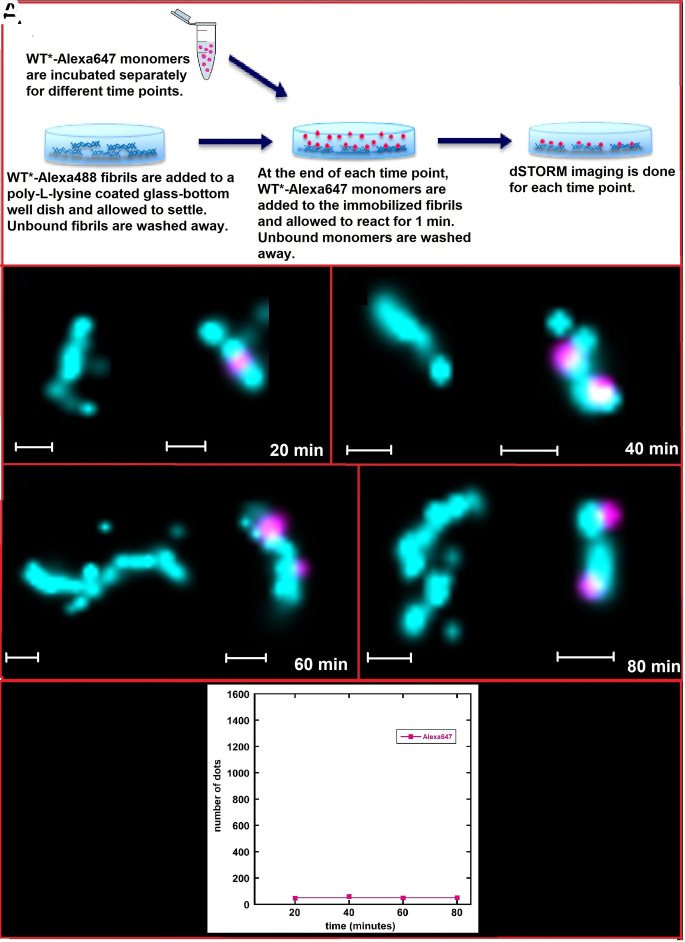
(*A*) dSTORM studies of fibrils after adding separately incubated monomer solutions. (*B*–*E*) WT*-Alexa647 monomers are incubated without preformed fibrils for 80 min and added to WT*-Alexa488 fibrils at 20-min intervals. Images are taken after 1-min incubation and subsequent washing. WT* monomers are labeled with Alexa647 (fuchsia) and WT* fibrils are labeled with Alexa488 (cyan). The scale bars represent 200 nm. For each time point, the dSTORM experiments were repeated three times. (*F*) Semiquantitative analysis of Alexa647 signal derived from WT* A*β*42 monomers in terms of the number of fluorescence dots appearing on the fibrils. Error bars represent the SD of Alexa647 signal from different image frames at each time point. The scale of the *y*-axis is the same as the scale of the *y*-axis in Fig. 3*J*.

The dSTORM images (Fig. 3 and *SI Appendix*, Fig. S1) do not distinguish between monomer clusters that nucleate and grow on the surface of fibrils from structures that grow in solution and then associate with the fibril seeds in a second step. To resolve this question, the experiment using separate fluorophores in seeds and monomers was repeated with the monomers incubated in the absence of the seeds for different times before being added to the seeds. WT*-Alexa647 monomers were thus incubated at 0.7 μM in 200 μL of 20 mM sodium phosphate, 0.2 mM EDTA, pH 8.0, without preformed seeds for 20, 40, 60, or 80 min, after which the solution was added to WT*-Alexa488 fibrils immobilized on a poly-lysine-coated # 1.5 (0.17 mm) glass-bottom dish (200 μL volume and 0.7 μM concentration). After incubating with the seeds for 1 min, any unbound species were washed away and the glass-bottom dish was loaded onto the dSTORM for imaging (Fig. 4). At all time points, the fibrils are free from any extended structures of opposite color along their sides. Some monomer clusters can be seen at various time points, but we do not observe any growth in size of these clusters as the reaction progresses. This is a strong indication that while some clusters have formed in solution, and these clusters can attach to fibril surfaces, most of the monomer aggregation and growth along the fibril sides is driven by the catalytic nature of fibril surfaces during seeded aggregation reactions.

Small dots from Alexa647 (WT* monomer) signals from dSTORM images were counted using ImageJ software. Fig. 4*F* shows the average number of dots derived from monomers near fibril surface in pink. Each dot can be counted as one molecule of A*β*42. The number of A*β*42 monomers at the fibril surface does not increase throughout all time points.

## Cross-Seeded Aggregation Kinetics Monitored by dSTORM

It has been previously shown that WT monomers fail to nucleate on 4S A*β*42 seeds (16). For this reason, we used 4S*-Alexa488 fibrils for cross-seeding studies to compare how WT monomers interact with fibril surfaces that are not catalytic to their proliferation.

**Fig. 5.**
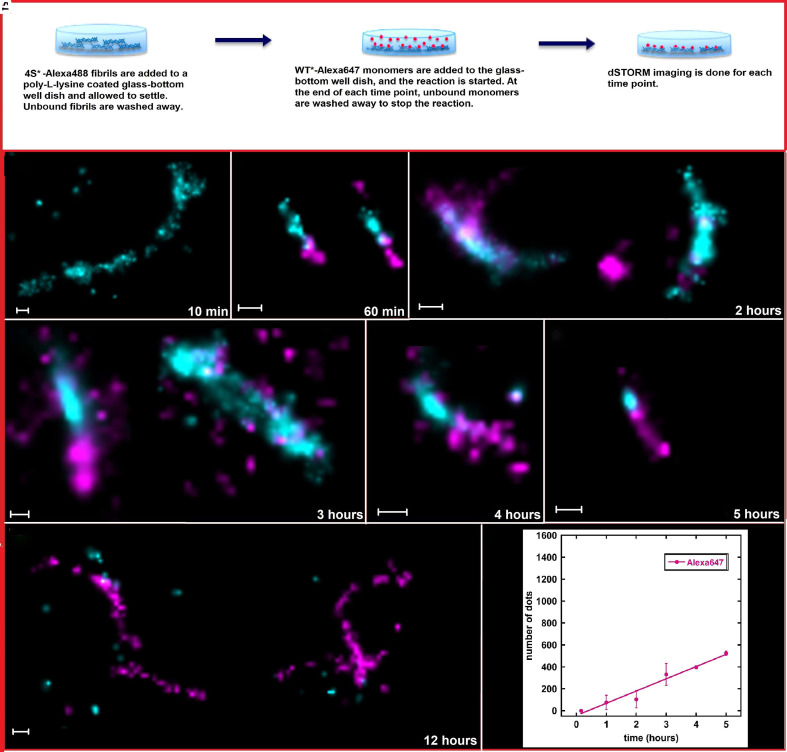
(*A*) Schematic representation of cross-seeded aggregation studies to follow surface catalysis of WT* A*β*42 monomers on 4S* fibrils using dSTORM imaging. (*B*–*H*) The cross-seeded aggregation reaction was followed using time points at 60-min intervals up to 12 h. WT* monomers are labeled with Alexa647 (fuchsia), and 4S* fibrils are labeled with Alexa488 (cyan). The scale bars represent 200 nm. For each time point, dSTORM experiments were repeated three times. (*I*) semiquantitative analysis of Alexa647 signal derived from WT* A*β*42 monomers. Error bars represent the SD of Alexa647 signal from different image frames at each time point. The scale of the *y*-axis is the same as the scale of the *y*-axis in Fig. 3*J*.

For studies of cross-seeding aggregation reactions, 4S*-Alexa488 fibrils (200 μL volume and 0.7 μM concentration) in 20 mM sodium phosphate and 0.2 mM EDTA, pH 8.0, buffer were deposited on a poly-lysine-coated glass-bottom dish. Once the fibrils were immobilized, WT*-Alexa647 monomers were added (200 μL volume and 0.7 μM concentration) to the fibrils for the reaction to begin. At the end of each time point, the unbound monomers were washed away in order to stop the reaction, and the glass-bottom dish was loaded onto the dSTORM microscope for imaging (Fig. 5*A*). Fig. 5 *B*–*H* shows the dSTORM images of various time points taken during the cross-seeding reaction of WT* A*β*42 monomers on 4S* A*β*42 fibrils. At the first time point *t* = 10 min, we see 4S* fibrils (cyan) alone with no monomers present. No interaction between WT* monomers and 4S* fibrils can be noted until *t* = 60 min, where small monomer clusters can be seen growing at the ends of fibrils. At *t* = 2 h, WT* monomers can be seen on the surface along the fibril as well as growing at the ends of fibrils. The monomers at the fibril surfaces fail to grow into larger aggregates but can elongate the 4S* seeds, as can be seen at *t* = 3 h. At 4 h and 5 h, the 4S* fibril can be seen growing via elongation by WT* monomers. Notably, the growth is highly polarized at one end of the fibril. When left for 12 h, the WT* monomers have formed long fibrils of their own, which have attached to the poly-lysine-coated surface of the dish, and hence can be detected in dSTORM images.

Small dots from Alexa647 (WT* monomer) signals from dSTORM images were counted using ImageJ software. Fig. 5*I* shows the average number of dots derived from monomers at fibril ends in pink. Each dot can be counted as one molecule. At the initial time point, no monomers are detected. Over the next time points, 4S* fibrils are elongated gradually by monomer growth, as can be seen by the linear rise in the number of dots over 2, 3, 4, and 5 h time points.

### Competition of Monomers for WT* and 4S* Fibrils.

**Fig. 6.**
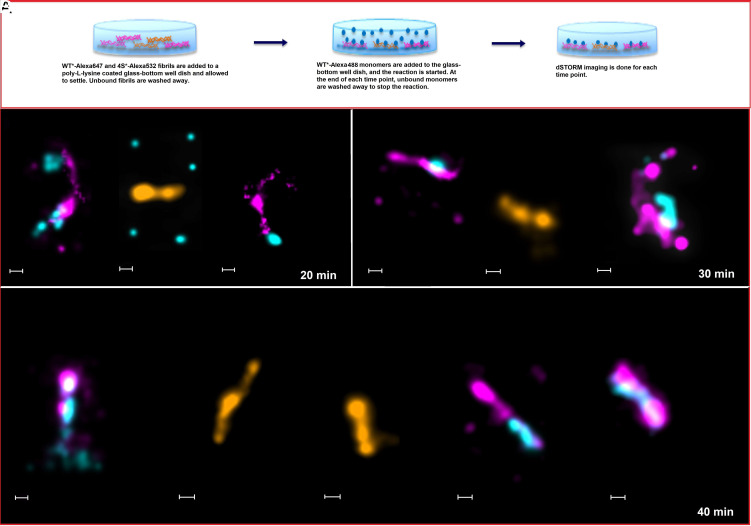
(*A*) Schematic representation of seeded aggregation studies to follow competitive binding of WT* A*β*42 monomers to WT* and 4S* fibrils using dSTORM imaging. (*B*–*D*) The seeded aggregation reaction to study competitive binding was followed using time points at 10-min intervals up to 40 min. WT* monomers are labeled with Alexa488 (cyan), WT* fibrils are labeled with Alexa647 (fuchsia), and 4S* fibrils are labeled with Alexa532 (orange). The scale bars represent 200 nm. For each time point, dSTORM experiments were repeated three times.

To study whether there is competition in binding of WT* monomers to WT* fibrils and 4S* fibrils, we performed binding experiments wherein monomers were allowed to interact with both types of fibrils simultaneously to study which interaction is preferred. WT* A*β*42 monomers were labeled with Alexa488, and WT* A*β*42 fibrils were labeled with Alexa647, while the 4S* fibrils were labeled with Alexa532. WT* A*β*42-Alexa647 and 4S*-Alexa532 fibrils (mixed 1:1 in 200 μL volume and total concentration of 0.7 μM) in 20 mM sodium phosphate and 0.2 mM EDTA, pH 8.0, buffer were deposited on a poly-lysine-coated glass-bottom dish. Once the fibrils were immobilized, WT*-Alexa488 monomers were added (200 μL volume and 0.7 μM concentration) to the fibrils for the reaction to begin (Fig. 6*A*). At the end of each time point, the unbound species were washed away in order to stop the reaction, and the glass-bottom dish was loaded onto the dSTORM for imaging. Fig. 6 shows the dSTORM images of various time points taken during the seeding reaction of WT* A*β*42 monomers on the mixture of WT* and 4S* A*β*42 fibrils. For technical reasons, monitoring of only two fluorophores at a time was possible. Hence, larger field views were first used to identify the locations of WT* fibrils and 4S* fibrils present in the same glass-bottom well dish. We then zoomed in on those fibrils separately and first recorded dSTORM images for WT* monomers with WT* fibrils and subsequently WT* monomers with 4S* fibrils.

No growth is observed along the labeled 4S* fibrils (orange) during all time points (Fig. 6 *B*–*D*). However, we observe at the first time point *t* = 20 min a few clusters of WT* monomers (cyan) along the surfaces of WT* fibrils (fuchsia). The same trend is seen at time point *t* = 30 min, where labeled monomer clusters along the WT* fibrils seem to have grown larger, and at *t* = 40 min, large clusters of monomers are seen along the length of the WT* fibrils. This follows the trend of monomer interactions with fibrils seen during self-seeding experiments of WT*. This competition experiment supports the idea that WT* monomers preferentially bind to WT* fibrils and grow along their length, while 4S* fibrils do not present any competition to the binding of monomers, when present in the same sample as the WT* fibrils.

## Discussion

The effect of various intrinsic factors on secondary nucleation of A*β*42 has been extensively studied during the recent past. Hydrophobicity (16), net charge of peptide, and size of side chains (23) all seem to affect the rate of secondary nucleation to varying extents. Secondary nucleation does not seem to be reliant exclusively on any one of these factors but rather seems to be a robust property that can persist sequence and structural perturbations. The comparison of self- and cross-seeding studies is a fruitful route toward understanding the effects of various intrinsic factors in surface-catalyzed secondary nucleation as well as elongation.

In this study, we used dSTORM to compare self- and cross-seeding of A*β*42. We aimed to probe the difference in behavior of A*β*42 monomers when interacting with, on the one hand, fibrils that catalyze their nucleation, and on the other hand, fibrils that show no catalytic effect (16). For this purpose, we use Alexa-labeled S8C A*β*42 as a model for WT A*β*42 (WT*), since this variant and labeling position is known to not cause any significant perturbations to fibril morphology or aggregation kinetics, and can aggregate independently without the presence of any unlabeled peptide (22). We successfully labeled the Alexa488 fluorophore to a mutant form of the 4S peptide with cysteine at position 40. As observed in ref. 22, covalent attachment of Alexa488 at this position in A*β*42 does not cause significant perturbations in aggregation kinetics and fibril morphology. The Alexa488-labeled 4S* fibrils served as seeds for the cross-seeding experiments. We labeled monomers with Alexa647 and fibrils with Alexa488 for both self- and cross-seeding experiments. Having the reacting species (monomers) and the catalytic surface (fibrils) labeled with two different fluorophores helps us gain insights into the nucleation process.

When WT* A*β*42 monomers are provided with WT* A*β*42 seeds, the monomers are observed to adhere to the fibril surface in drastically less time (∼10 min) as compared to 4S* fibrils (60 min). There is a period of slow growth where small clusters of monomers gather on the fibril surface, followed by a period of faster growth where the small monomer clusters are converted to larger aggregates on the fibril surface. This correlates with the conventional ThT fluorescence assays used to follow amyloid aggregation, which show a sigmoidal growth curve with exponential growth close to the midpoint of the macroscopically observable transition. Most of the larger aggregates, which get detached from the parent fibril, are washed away at the end of the reaction, but some aggregates can be detected as individual fibrils from 50 min and onward (Fig. 3). These fibrils are short, indicating that their formation depends on a high rate of secondary nucleation. Further, we can see that WT* monomers can form clusters in solution, which may adsorb on fibril surfaces in a second step. However, the aggregation and formation of elongated structures along the length of fibrils are dominated by processes at the fibril surface, most likely involving the surface-catalyzed nucleation on fibril surfaces. In the cross-seeded aggregation, the main process contributing to monomer growth seems to be growth at the 4S* fibril ends steadily over a period of time (Fig. 4). This elongation by monomers on fibrils can be seen to be polarized at one end of the fibril, which agrees with previous studies performed specifically to study A*β*42 elongation, albeit using synthetic peptides (24). The WT* monomers are seen to interact on the 4S* fibril surface but fail to convert into larger aggregates which can detach from the parent fibrils. This points to the fact that while WT* monomers will come in contact with the 4S* seed fibrils, the contact events fail to lead to aggregation and growth along the fibril surfaces when monomers cannot adopt the parent fibril structure.

When surface-catalyzed secondary nucleation drives the aggregation process, a clear exponential period of growth is noticed, after an initial lag phase (Fig. 3*J*). However, in the case of 4S* fibrils, the monomers are only able to elongate the fibrils and unable to grow on their surfaces. As a result, growth from monomers is linear (Fig. 4*F*). The drastic difference in time scale and evolution of the self-seeding and cross-seeding reactions indicates a difference in rates of secondary nucleation–dominated growth as compared to elongation. Competitive binding studies using fibrils of two separate colors (Fig. 6) confirm that 4S fibrils cannot compete with WT fibrils for binding and growth of WT monomers when present in the same solution, showing that monomers preferentially bind to and grow on fibrils of the same fold, on which they can take up the parent fibril structure.

It is an apparent paradox that an amyloid protein may on the one hand undergo efficient nucleation on amyloid fibrils of a different protein (25) or on a foreign surface such as polymeric nanoparticles (26–28), while, on the other hand, cross-nucleation is hindered in some cases with high sequence similarity (16, 17). A striking example is peptides with identical sequence but opposite chirality, the fibrils of which completely fail to cross-catalyze the nucleation of monomers of opposite handedness (29). These fibrils are assembled from sequences of identical hydrophobicity and charge distribution but of opposite amino acid chirality. Thus, while general hydrophobicity and other surface properties can support heterogeneous primary nucleation, the catalytic processes underlying secondary nucleation may have a much higher level of structural specificity. Another striking example is C-terminal length variation, in which case A*β*42 fibrils fail to catalyze the aggregation of A*β*40 monomers, and vice versa, related to the two alloforms having very different fibril folds (17). In contrast, N-terminally extended peptides that share the fibril core sequence of A*β*42 are fully compatible in cross-seeding with A*β*42 (30). The present case belongs to the category of high sequence similarity but a variant fibril fold; the 4S fibrils have been shown by cryo-TEM to fold in a different way compared to A*β*42 wild-type, and again, cross-catalysis of surface nucleation fails (16, 17).

These findings together imply an underlying molecular selectivity in fibril-catalyzed nucleation, which will only be at hand in cases where the nucleating monomers can fold in the same way as the monomers in the fibril structure. The current dSTORM results add to this view by showing that monomers may adhere to fibrils of a variant fold, whereas nucleation and growth are only observed along fibrils of a compatible fold. This is most likely due to a structural frustration; if the monomers try but fail to adopt the fold of a variant peptide, which is disfavored by steric repulsion, repulsive electrostatic interactions, or other unfavorable interactions, this will result in dissociation from the seed rather than nucleation and growth along the seed.

## Conclusion

In conclusion, the findings of this two-color dSTORM study clearly show that monomer interaction and growth along fibril surfaces are dependent on the ability of the fibril surface to catalyze nucleation. When there is structural compatibility between monomers and fibrils, the monomers nucleate on the fibril surface and grow into larger aggregates before detaching as independent aggregates. This represents an exponential period of the aggregation process, under which the reaction progresses very fast. In contrast, when the monomers cannot copy the structure of the parent fibril, the monomers interacting with fibril surfaces fail to nucleate, and the aggregation reaction proceeds as slowly as in the absence of seeds. No growth along the sides of fibrils is observed in this case. Together, our finding gives further impetus to the view that surface-catalyzed secondary nucleation is strongly linked to the ability of the monomers to adopt the parent fibril structure, whereas peptide variants that would suffer from steric repulsion, repulsive electrostatic interactions, or other unfavorable interaction if folded the same way as the seed fibril fail to nucleate and grow along the seed.

## Materials and Methods

### Expression and Purification of Peptides.

The plasmid carrying synthetic genes with *E. coli* optimized codon for A*β*42 S8C (pET3a, purchased from GenScript) was transformed into Ca^2+^ competent cells of *E. coli* strain BL21 DE3 pLysS star, and the protein was expressed in autoinduction medium (22, 31). The peptide was purified using ion exchange chromatography (IEX) as described (32, 33) with the minor change that lower salt concentration (50 mM NaCl) was used to elute the peptides, and size exclusion chromatography (SEC) on a 26- × 600-mm Superdex 75 column was used instead of spin filters for molecular mass fractionation. The ion exchange and SEC buffers also contained 1 mM dithiothreitol (DTT) to avoid dimerization of cysteine mutants as described (22). The final SEC was performed using buffers without DTT. The purified monomeric peptide was lyophilized as aliquots until further use.

### Expression and Purification of EDDIE-Tagged Construct.

4S A*β*42 and 3S-V40C A*β*42 were expressed using a fusion construct with the self-cleavable tag EDDIE (11). The plasmids carrying synthetic genes with *E. coli* optimized codon for EDDIE-A*β*42 4S and EDDIE-A*β*42-3S-V40C (pET3a, purchased from GenScript) was transformed into Ca^2+^-competent cells of *E. coli* strain BL21 DE3 pLysS star, and the proteins were expressed in autoinduction medium (31). The cell pellet from 1.5-L culture was sonicated five times in 10 mM Tris HCl and 1 mM EDTA, pH 8.5, with a trace of DNase, 50 mL each time. After each sonication, the lysate was centrifuged for 7 min at 15,000 rpm (5534.1×*g*) and the supernatant removed. The inclusion body pelleted after the fifth sonication was dissolved in 70 mL 10 M urea, 10 mM Tris, 1 mM EDTA, and 1 mM dithiothreitol (DTT), pH 8.5, by sonication and stirring. When dissolved, the urea solution was diluted with 80 mL 10 mM Tris, 1 mM EDTA, and 1 mM DTT, pH 8.5, and loaded onto 3× 5-mL DEAE-FF columns in tandem. Before this, the column was preequilibrated in 10 mM Tris, 1 mM EDTA, and 1 mM DTT, pH 8.5, with 4 M urea. The column was washed with 100 mL 4 M urea, 10 mM Tris, 1 mM EDTA, and 1 mM DTT, pH 8.5, and eluted with a linear gradient from 0 to 0.4 M NaCl in 4 M urea, 10 mM Tris, 1 mM EDTA, and 1 mM DTT, pH 8.5 (180-mL gradient). The fractions were collected using a fraction collector, analyzed by sodium dodecyl sulfate polyacrylamide gel electrophoresis (SDS/PAGE), and pooled together into one or more pools depending on their purity. Each pool was diluted 15 times with 10 mM Tris, 1 mM EDTA, and 5 mM DTT, pH 8.5, in a glass bottle, placed in a cold room, and left there for 48 to 72 h to cleave off the EDDIE tag. Cleavage was monitored using SDS/PAGE. The peptides were then purified from EDDIE using IEX in batch format on Q-Sepharose big beads and eluted with 50 mM NaCl, lyophilized, dissolved in 6 M GuHCl, 20 mM sodium phosphate, and 0.2 mM EDTA, pH 8.5, and further purified using SEC on the 26- × 600-mm Superdex 75 column. The monomer fractions were lyophilized as aliquots until further use.

### Labeling of Peptides with Alexa Fluor.

Lyophilized fractions (∼14 μM) of the peptides were dissolved in 50 μL Milli-Q water. Alexa fluor 488 or Alexa fluor 647 of concentration 3 to 4 mM dissolved in 20 μL dimethyl sulfoxide (DMSO) was added to the dissolved peptide in order to have excess dye in the labeling mix and kept overnight at 4 °C for labeling. The following morning, the mix was added in 1 mL of 6 M GuHCl, 20 mM sodium phosphate, 0.2 mM ethylenediaminetetraacetic acid (EDTA), pH 8.5, and subjected to gel filtration on a Superdex 75 10/300 column in 20 mM sodium phosphate buffer, pH 8.0, with 0.2 mM EDTA. The absorbance at 214 nm, 280 nm, and 488 nm or 647 nm was monitored using Quadtech detectors to follow the elution of the labeled peptide and to monitor any unlabeled peptide, if present. The aliquots collected from the SEC were analyzed by SDS PAGE and stored at −80 °C until further use.

### Preparation of Fibrils of Alexa-Labeled Peptides.

For Alexa488-labeled S8C A*β*42, 5.5 μM monomers in 20 mM sodium phosphate and 0.2 mM EDTA, pH 8.0 buffer were kept for aggregation in a 96-well plate (Corning 3881), 100 μL per well. The experiments were initiated by placing the 96-well plate at 37 °C in a plate reader (Clariostar Omega). The quenching of Alexa488 was followed through the bottom of the plate every 120 s. The excitation filter was at 488 nm, and the emission filter was at 519 nm.

For Alexa488-labeled 3S-V40C, aggregation reactions were performed for 0:1, 1:1.5, 1:3.5, 1:7, 1:15, 1:23, 1:31, and 1:38 ratios of peptides (3S-V40C-Alexa488: 4S) in 20 mM sodium phosphate and 0.2 mM EDTA, pH 8.0, buffer with 6 μM Thioflavin t (ThT). The final total monomer concentration was close to 5 μM. The aggregation reaction mix was pipetted into a 96-well plate (Corning 3881), 100 μL per well. The experiments were initiated by placing the 96-well plate at 37 °C in a plate reader (Fluostar Omega). The ThT fluorescence was measured through the bottom of the plate every 120 s. The excitation filter was at 440 nm, and the emission filter was at 480 nm.

The fibrils formed were aliquoted before storing at −80 °C to avoid repeated freeze–thaw cycles.

### Seeded Aggregation Kinetics Monitored by dSTORM.

To study self- and cross-seeding kinetics by dSTORM, 200 μL of 0.7 μM Alexa-488-labeled fibrils were deposited on a poly-lysine-coated # 1.5 (0.17 mm) glass-bottom dish (WillCo Well, Germany). A low concentration of fibrils is necessary to be able to observe individual fibrils. The fibrils were allowed to settle for 20 min and washed with 200 μL 20 mM sodium phosphate and 0.2 mM EDTA, pH 8.0, buffer. Seeding kinetics were studied at the following time points: 200 μL of 0.7 μM Alexa-647-labeled monomers was added to the immobilized fibrils on the glass-bottom dish at *t* = 0 for the reaction to begin. At the end of each time point, free monomers were washed off with 200 μL 20 mM sodium phosphate and 0.2 mM EDTA, pH 8.0, buffer to stop the reaction. The glass-bottom dish was then loaded onto the microscope. A separate glass-bottom dish was set up for each of 8 different time points, and dSTORM imaging was done three times for each dish. Additionally, each time point was set up with reversed labels, thus each time point of each seeding reaction was imaged at least 6 times, and there are in total 48 images taken along each reaction. The number of repeats of each experiment can be found in *SI Appendix*, Table S1.

dSTORM imaging was performed on the ELYRA P1 imaging system (Zeiss, Germany), which included an inverted microscope with a 100X oil immerse objective lens (1.46 NA). The samples were mounted in the ZEISS level-adjustable insert holder and placed on the PIEZO stage with Auto-focus adjusted. The fluorescence dyes were excited by the selected three laser lines, 488, 543, and 633 nm, respectively. Accordingly, the filter sets #4 for collection of the emission lights were chosen dependent on the fluorescence dyes. For Alexa 488, the 488-nm laser line was used for excitation and the emission light filtered BP 495–550; for Alexa 546, a 543-nm laser line was used for excitation and the emission light filtered BP 570–620; for Alexa 647, a 633-nm laser line was used for excitation and the emission light filtered LP 655. The images were acquired onto a 256 × 256 pixel frame of an electron multiplying charge coupled device (EMCCD) camera (iXon DU897, Andor).

To generate the dSTORM images, the PALM processing function in ZEN software was applied. First, the overlapping signals were discarded by using a multiemitter model for the whole image sequences. Second, to distinguish the real signal peak, the mask size was set to 7 pixels, and the ratio of signal/noise was set to 7. After the filtration, the lateral and axial drift during acquisition was corrected after reconstruction of dSTORM images by using the Drift function that was tested by fluorescent beads as a fiducial marker prior to the analysis. After drift correction, the images were proceeded by grouping function. Finally, the present dSTORM images were corrected according to the distributions of the following parameters: photon number, precision size, and first frame. To remove the unspecific background, the molecules were filtered out if the number of molecules was less than 10 in an area of 100-nm perimeter.

### Cryo-TEM.

For 4S A*β*42, a ratio of 1:1.5 (labeled 3S-V40C:unlabeled 4S) peptide with the total monomer concentration close to 5 μM was incubated at 37 °C in PEGylated plates (Corning 3881) in a plate reader and collected after reaching the plateau in ThT fluorescence. Specimens for cryo-TEM were prepared in an automatic plunge freezer system (Leica EM GP). The climate chamber temperature was kept at 21 °C, and relative humidity was 90% to minimize loss of solution during sample preparation. The specimens were prepared by placing 4 μL solution on glow-discharged lacey formvar–carbon-coated copper grids (Ted Pella) and blotted with filter paper before being plunged into liquid ethane at −183 °C. This leads to vitrified specimens, avoiding component segmentation and rearrangement, and the formation of water crystals, thereby preserving original microstructures. The vitrified specimens were stored under liquid nitrogen until measured. A Fischione Model 2550 cryotransfer tomography holder was used to transfer the specimen into the electron microscope, JEM 2200FS, equipped with an in-column energy filter (Omega filter), which allows zero-loss imaging. The acceleration voltage was 200 kV, and zero-loss images were recorded digitally with a TVIPS F416 camera using SerialEM under low-dose conditions with a 10 eV energy–selecting slit in place.

## Supplementary Material

Appendix 01 (PDF)

## Data Availability

Raw images for data shown in Figs. 5 and 6 have been deposited in Figshare (https://doi.org/10.6084/m9.figshare.20392974; https://doi.org/10.6084/m9.figshare.20392968).
