# Supplementary material for: Direct observation of secondary nucleation along the fibril surface of the amyloid β 42 peptide
Source: Proc Natl Acad Sci U S A. 2023 Jun 12;120(25):e2220664120. doi: 10.1073/pnas.2220664120 (PMC10288637; doi:10.1073/pnas.2220664120)
Supplement: Supplementary file 1 — Appendix 01 (PDF) [file pnas.2220664120.sapp.pdf]

Supporting Information

Table S1: Number of individual experiments and images for each figure.

Figure number	Independent Experiments	dSTORM images
Figure 3	8	24
Figure 4	4	12
Figure 5	7	21
Figure 6	3	9
Figure 7	3	9

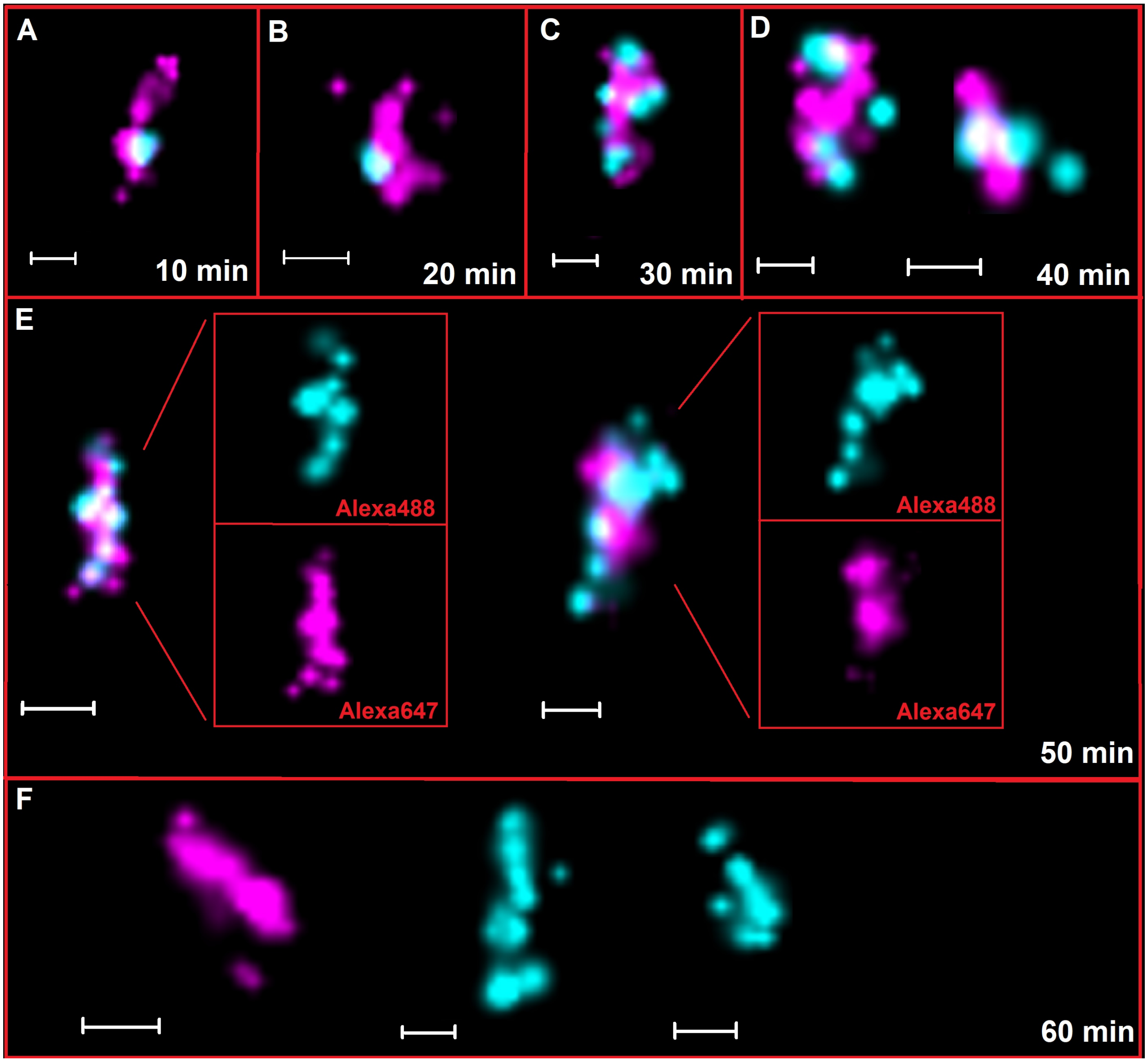

Figure S1: (A-F): Self-seeded aggregation studies to follow surface catalysis of WT* Aβ42 using dSTORM imaging. The aggregation reaction was followed over time, with 10 min intervals up to 60 min. WT* monomers are labelled with Alexa488 (cyan) and WT* fibrils are labelled with Alexa647 (fuchsia). The scale bars represent 200 nm. For each time point, dSTORM experiments were repeated three times.

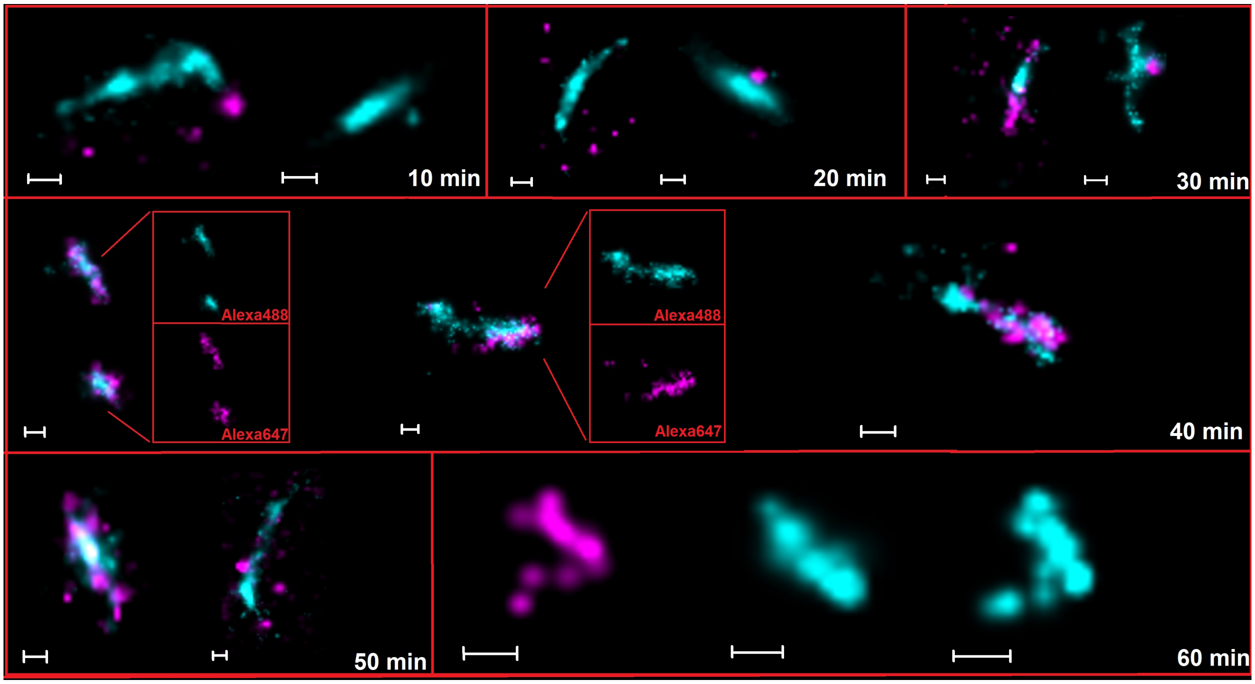

Figure S2: Data from all self-seeded aggregation studies from Figure 3 to follow surface catalysis of WT* Aβ42 using dSTORM imaging. The aggregation reaction was followed over time, with 10 min intervals up to 60 min. WT* monomers are labelled with Alexa647 (fuchsia) and WT* fibrils are labelled with Alexa488 (cyan). The scale bars represent 200 nm.
